# Bias and Beliefs: Age Discrimination, Perceived Control over Cognitive Aging, and Executive Functioning in Caregiving Grandparents

**DOI:** 10.3390/geriatrics11040087

**Published:** 2026-07-15

**Authors:** Maia A. McLin, Laura A. Shillingsburg, Amara L. Mason, Kourtney Mia Barfield, Danielle Kristen Nadorff

**Affiliations:** Department of Psychology, Mississippi State University, Mississippi State, MS 39762, USA; mam1242@msstate.edu (M.A.M.);

**Keywords:** cognition, executive function, discrimination, perceived control over cognitive aging, caregiving grandparents

## Abstract

**Background:** Caregiving grandparents face unique stressors that may affect cognitive health. Discrimination is associated with poorer cognitive performance, whereas stronger perceived control over cognitive aging is associated with better performance. This study examined how everyday discrimination and perceived control over cognitive aging relate to executive function in caregiving grandparents. **Methods:** Using cross-sectional data from the Midlife in the United States study (MIDUS 3), we analyzed 1326 grandparents (166 caregiving and 1160 non-caregiving) with complete data on all study measures. Everyday discrimination, perceived control over cognitive aging, and executive function were assessed with self-report surveys and telephone-based cognitive assessments. Mediation and moderated mediation models were estimated with covariates. **Results:** Perceived control over cognitive aging mediated the relation between discrimination and executive function, and this mediation was moderated by caregiving status. Among non-caregivers, stronger perceived control was associated with a weaker negative relation between discrimination and executive function. This protective pattern was not observed among caregiving grandparents. The moderation was attenuated to nonsignificance when an expanded covariate set was added, so it should be considered preliminary. **Conclusions:** The demands of caregiving may lessen the influence of control beliefs on cognitive performance. Because the design is cross-sectional, these results describe statistical rather than temporal mediation. The findings support targeted interventions for this vulnerable population, although they warrant further investigation.

## 1. Introduction

Aging is the biggest risk factor for neurocognitive disorders. In the United States, Baby Boomers born before 1965, making them 60 years of age or older, are about 20% of the population and are at higher risk for developing Alzheimer’s disease and related disorders (ADRDs) [1]. Informal and unexpected caregivers over age 65 make up a significant part of the caregiving population (e.g., custodial grandparents, multigenerational home grandparents, ADRD caregivers, chronic and/or terminal illness caregivers), around 34% [2]. This population is more likely to report memory concerns than non-caregivers, which may not directly relate to cognitive function but can influence behaviors that contribute to cognitive function, such as participating in cognitively stimulating activities [2]. Cognitive health is a public health issue because the prevention of cognitive decline, which may lead to ADRDs, is possible, and researchers have identified social and biological determinants that may be mitigated to improve cognitive health and potentially delay or prevent the development of ADRDs. Current prevention measures for ADRDs have been established by the UsAgainstAlzheimer’’s workgroup, which determined 6 areas that clinicians should educate patients 45 years of age and older about in terms of cognitive decline. This list was not exhaustive and included neurovascular risk management, physical activity, sleep, nutrition, social activity, and cognitive stimulation [3]. Older adults who are caregivers for their grandchildren are a population with unique stressors and risk factors that could affect their cognitive abilities. These grandparents face challenges such as social and economic disadvantages, potential for discrimination, and the added burden of caregiving, which can contribute to their likelihood of experiencing cognitive decline [4,5,6,7,8,9]. Despite these challenges, some factors may protect against cognitive decline in caregiving grandparents. One such factor is their perceived control over cognitive aging. It is important to investigate how risks and protective factors interact and influence cognitive outcomes within this population to provide additional education and intervention to protect cognitive health [10]. Within the Stress Process framework, experiences of daily discrimination can be conceptualized as a primary stressor that undermines cognitive functioning, whereas caregiving for grandchildren represents a chronic secondary stressor that may amplify overall stress burden. Perceived control over cognitive aging functions as a psychosocial coping resource that can buffer the impact of stressors on cognitive outcomes. However, when stress exposure is prolonged or compounded (as is often the case for caregiving grandparents), the protective effects of such resources may be diminished or overridden.

This study examines how being a caregiving grandparent shapes the associations among perceived discrimination, perceived control over cognitive aging, and executive function in older adults. Clarifying these connections can point toward better ways to support caregiving grandparents and protect their cognitive health [11,12].

Discrimination, broadly defined as the differential and unfair treatment of individuals based on perceived group membership, is a well-documented psychosocial stressor with adverse effects on physical, mental, and cognitive health [13,14]. Everyday experiences of discrimination (such as being treated with less courtesy, being followed in stores, or being assumed to be less intelligent) accumulate across time and contribute to allostatic load through repeated activation of the stress response. Among older adults, discrimination has been linked to poorer executive function, faster cognitive decline, and greater memory concerns [12,15,16]. Because caregiving grandparents occupy multiple socially devalued statuses (older adult, frequently low-income, often female, and sometimes racial or ethnic minority), they are particularly likely to encounter discrimination and to carry its cumulative toll on cognitive health. Recent population-based research links everyday discrimination to poorer cognitive function in middle-aged and older adults [17].

How individuals conceptualize discriminatory experiences also matters. Discrimination can be understood through a unidimensional framework, in which a person attributes unfair treatment to a single identity characteristic, or through an intersectional framework, in which the “multiplying” effects of overlapping marginalized identities shape both the experience and its interpretation [18]. The present study adopts an intersectional perspective, treating everyday discrimination as a composite stressor whose meaning and impact cannot be reduced to any single demographic category. This framing is especially relevant for caregiving grandparents, whose identities typically converge across age, gender, socioeconomic, and, for some, racial or ethnic lines. Beyond its direct physiological toll, discrimination also shapes psychological resources, including the beliefs older adults hold about their own aging, which we describe next.

### 1.1. Theoretical Background

Stress affects aging cohorts differently across the lifespan, with older adults being more sensitive to its effects [19]. Hormonal differences across the lifespan largely determine how stress affects an individual [20]. Women tend to experience more dysfunctional stress regulation at older ages due to ovarian deterioration [20]. Estrogen exposure after menopause increases sensitivity to stress, meaning that older adults who receive estrogen hormone treatments are at increased risk for stress-related effects, including neuropsychiatric conditions [20]. Because grandparent caregivers are more likely to be women and older adults, it is necessary to understand the influence of stress on biological systems and mental health. Both Stress Process Theory and Family Stress Theory have been used to explain how stressors influence caregiver outcomes. Stress Process Theory is a conceptual framework for recognizing that different processes occur because of an individual’s social status and that these processes are connected to outcomes in physical and mental health [21]. This theory is relevant to those with marginalized or nondominant social statuses, such as grandparent caregivers, as it recognizes the effect that additional stressors, such as discrimination or caregiver burden, can have on an individual’s well-being by affecting other life practices or beliefs.

This theory posits that stress-related outcomes are influenced by background factors and primary and secondary stressors. It also incorporates the influence of protective factors that can attenuate these stress-related outcomes. Stress Process Theory has been applied to caregiving grandparents to understand how coping influences outcomes, including depression and anxiety [22]. In these models, specific contextual factors include the type of relationship between the caregiver and care recipient and cultural norms. The primary stressor of caregiving can lead to secondary stressors, such as financial difficulties and loss of social support. As a result of caregiving, the grandparent also undergoes a role change and experiences a different social context. Therefore, caregiving grandparents may be more sensitive to stressors related to cognitive function, such as experiences of discrimination due to limited resources, the transition to the primary caregiver role, and a lower economic status typical of caregiving grandparents.

Family Stress Theory posits that a stressful event, such as a caregiving change, interacts with available resources (e.g., financial support, social support) to mitigate stress, and the interpretation of the event (e.g., how one copes) determines the outcome in the family [23,24]. Caregiving events may directly affect perceived control over cognitive aging, as caregiving grandparents often are not prepared to take on the role of providing care for a child. The effects of well-known stressors, such as discrimination, and the processes underlying these stressors on mental and physical health outcomes are often not studied among nontraditional family units, such as families where the primary caregivers are grandparents. In addition, older adults are particularly vulnerable to the effects of stress due to increased cortisol levels relative to younger adults, which can accelerate hippocampal atrophy and associated cognitive impairment [25]. Caregiving grandparents may face additional stressors compared with non-caregiving counterparts, which may reduce the influence of typical protective factors for certain stressors until the caregiving-related stresses are reduced (e.g., resources improve). This study investigates whether additional stressors, such as caregiving, can differentially affect processes, such as perceived control over cognitive aging, that underlie the effects of a stressor (i.e., discrimination) on cognitive function (i.e., executive function).

### 1.2. Cognitive Aging

Normal cognitive aging is important to differentiate from symptoms of mild cognitive impairment or Alzheimer’s disease and related dementias (ADRDs). Normal cognitive aging includes changes in conceptual reasoning, memory, and processing speed but does not result in functional impairment, whereas mild cognitive impairment or ADRDs are associated with functional impairment [26]. Decline in these abilities is often not noticeable and occurs throughout different developmental stages in life [27].

Memory and executive function decline are related to deficits in activities of daily living (ADLs). Memory impairment, specifically explicit memory, which consists of episodic and semantic memory, is often assessed to determine intellectual functioning and cognitive ability. Episodic memory, otherwise known as autobiographical memory, is memory of personal life events. This type of memory tends to decline over a lifetime. Thus, this decline is more likely to be noticeable earlier than the decline in semantic memory, which is related to learned knowledge and tends to increase with age until around 60 years of age, after which there is a subsequent incremental decline [27]. Deficits in executive function are reflected in the inability to plan, organize, and complete tasks. Executive functioning, while crucial to daily living, is especially important for caregivers of children raised in marginalized economic households, as caregiver executive function is related to the development of child executive function in this socioeconomic group and to the quality of the caregiver’s ability to function [28].

The ability to recognize these signs is important for access to resources that can slow cognitive decline and potentially delay the onset of dementia. Subjective assessment of cognitive function typically precedes objective assessment and serves as an early indicator of decline. Thus, the ability to recognize decline across various populations is crucial for providers and individuals seeking early intervention [29]. Studies have shown that individuals who experience more symptoms of poor mental health tend to have a worse ability to recognize cognitive changes [30]. Therefore, identifying reliable subjective measures of cognitive function that align with objective measures is vital among groups with additional stressors, such as caregiving grandparents.

Chronic life stress is associated with accelerated cognitive decline [7]. A population that receives little attention, is likely to experience chronic stress, reports poorer mental health outcomes, and is thus at elevated risk for cognitive impairment is custodial grandparents. Therefore, positive recognition of cognitive decline among caregivers may be confounded by higher levels of anxiety and/or depressive symptoms. Additionally, this population may be at higher risk for experiencing cognitive decline due to chronic stress.

### 1.3. Caregiving Grandparents

Grandparents may reside with their grandchildren for a variety of reasons, including serving as a custodial grandparent, living in a three-generational home, providing substantial care for their grandchildren, or potentially requiring care themselves. In 2021, around 2.1 million grandparents were primarily responsible for their grandchildren [9]. The most common reasons grandparents raise their grandchildren include death, disease, detention (incarceration), divorce, departure (parent migration), drugs, desertion (abandonment), delivery (teen pregnancy), deployment (military), dollars (need to share limited resources), duty, and daily grind (work responsibilities) [5,8]. These stressful circumstances affect both the grandparent’s well-being and the child in their care. Custodial grandparents often are not prepared to raise their grandchildren and, as a result, face financial strain, psychological stress, and poor mental health [31]. More specifically, custodial grandparents tend to report higher levels of stress, depression, and anxiety symptoms than non-caregiving older adults [4]. One aspect that custodial grandparents are more likely to encounter is caregiver burden. Caregiver burden is described as a multifaceted strain that can affect caregivers. Caregiving grandparents may experience unique stressors, such as additional financial insecurity, lack of support, poorer reported health, and conflict with the grandchild’s parents, compared to non-caregiving adults [6,32].

More common than custodial grandparents living without one or more of the child’s parents are co-residing grandparents who are primarily responsible for their grandchild or who live in a grandparent-maintained home while one or more of the child’s parents live in the household, which occurs about 80% of the time [9]. Multigenerational homes continue to increase in number overall in the United States. Co-residing grandparents in grandparent-maintained homes more commonly work themselves and provide support to their grandchildren through financial assistance, direct caregiving, and other support, compared to co-residing grandparents in parent-maintained homes [9].

Grandparents are now just as likely to be employed as retired while providing care for grandchildren, and their employment status may be affected by additional financial strain from caring for a child. Additionally, custodial grandparents and co-residing grandparents in grandparent-maintained homes are more likely to be considered economically marginalized [9], increasing the likelihood of interactions involving discrimination. This discrimination, if perceived as stressful, may have deleterious effects on mental and physical health [13]. When an individual perceives a discriminatory event as stressful, cortisol is released from the hypothalamic–pituitary axis (HPA). Persistent levels of cortisol in the body may lead to cognitive impairment [13].

There is a lack of research assessing cognitive function among various populations of custodial and co-residing grandparents. One study found that custodial grandparents have better verbal fluency and cognitive similarities and progress at a slower rate of cognitive decline compared to non-caregiving adults [33]. However, the participants in this study were limited to individuals who graduated from a particular Wisconsin school, and assessment of caregiving status as custodial was based on a question indicating that the grandparent had increased responsibility for a grandchild, not necessarily that they were primarily responsible for the grandchild’s care. Available resources for caregivers also have the potential to mitigate the effects of caregiving stress. Thus, the location of caregivers matters because regions in the United States differ in access to resources.

Another study found that caregiving grandparents reported higher performance on objective cognitive function, specifically word recall [34]. This study involved caregiving grandparents in South Africa. There are several differences in this population that may lead to less generalizable results, such as a higher expectation for grandparents to care for grandchildren due to the HIV/AIDS epidemic affecting much of the middle generation, resulting in the loss of the middle generation, and potential differences in aging stereotypes or beliefs about aging, resulting in less discrimination that acts as a stressor for this population.

One potentially protective component of cognitive function is perceived control over cognitive aging, closely related to locus of control [10,11,12]. However, this concept may not be as relevant for custodial grandparents or co-residing grandparents who contend with additional stressors and experiences that are outside their control. Therefore, it is important to consider custodial and co-residing grandparents as a culture to understand potential influences on their perceived control over cognitive aging and to determine whether this same mechanism may be predictive of cognitive function among this group.

### 1.4. Cultural Considerations

Cognitive brain health assessment of older adults is important to identify potential impairment that may affect activities of daily living and instrumental activities of daily living. A decline in these abilities can lead to health, safety, and financial risk. Caregivers tend to have higher rates of anxiety and depressive symptoms [4].

Previous studies have shown that those with more anxiety and depressive symptoms tend to be worse at recognizing deficits in cognitive ability [30]. Therefore, perceived control over cognitive aging may be less likely to align with objective cognitive assessment among caregivers because this population is more likely to experience anxiety and depressive symptoms as they contend with a higher proportion of everyday stressors than non-caregiving individuals.

Help-seeking behavior for cognitive impairment varies across populations, influenced by socioeconomic status, impairment severity, aging beliefs, memory knowledge, and experiences with healthcare providers [35]. Income inequality, by itself, is not a likely predictor of reporting subjective cognitive decline (SCD). However, self-ranking relative to others is more predictive of SCD reporting, with those of higher income ranking having lower odds of reporting SCD [36]. Another barrier to help-seeking is the consideration of brain health among older adults. Older adults are more likely to feel susceptible to cognitive decline if they have a family member with dementia, notice signs of cognitive decline, and know someone with cognitive decline [37].

### 1.5. Detecting Cognitive Decline

A variety of factors influence the likelihood of detecting cognitive decline, making it valuable to recognize how and what lifestyle differences affect caregiving and non-caregiving older adults’ recognition of cognitive decline. Recognition of memory decline is typically first apparent to the individual before it is recognized by family members [38].

Memory beliefs influence help-seeking behavior. These beliefs can include ideas about the possible deterioration of symptoms, the likelihood of their continued presence, control over memory performance, and whether one’s memory seems better or worse compared to others [39]. Individuals who report symptoms as due to memory, believe their memory is worse than peers’, believe there are consequences to their memory symptoms, and/or are more likely to seek help due to their concerns [39].

While evidence is contradictory regarding the relationship between recognizing cognitive decline and objective measures of cognitive function (i.e., episodic memory, executive function, verbal fluency), it is agreed that seeking medical care and early intervention when perceptions of cognitive decline occur are crucial for treatment in the presence of potential ADRDs or neurocognitive disorders.

Perceived discrimination affects the likelihood of reporting memory concerns, with those who report more daily discrimination also reporting more memory concerns, likely due to experiences of stereotype threat [16]. For example, an older adult who perceives a discriminatory event, such as not receiving a job due to their age even though they are the most qualified, may then hypothesize that they do not think as quickly or are not as smart as younger adults (stereotype threat), which reinforces their belief that their memory may be of concern. The higher likelihood of these reports among individuals who experience daily discrimination indicates that these concerns may not be linked to objective cognitive function [16]. This highlights the importance of considering social and environmental factors, such as perceived discrimination, when assessing memory concerns in older adults.

Caregiving grandparents, who tend to report more stressors than non-caregiving grandparents [40], may also report higher levels of memory concerns that are not related to objective cognitive testing. Despite having more memory concerns, those in the caregiving role are less likely to seek help for psychological and physical concerns [41]. Thus, more research is needed on the risk and protective factors related to cognitive decline among this understudied population.

### 1.6. Perceptions of Discrimination

How individuals conceptualize experiences of discrimination can vary. They may perceive discriminatory actions through unidimensional or intersectional frameworks. Those who perceive discrimination within a unidimensional framework may have multiple marginalized identities but attribute the perceived discrimination to only one aspect of their identity. For example, a man may identify as queer, Black, and having cerebral palsy but attribute experiences of discrimination to one aspect of their identity, such as their gender identity. Another common framework for perceiving discrimination is an intersectional approach. Those who perceive discrimination within an intersectional framework are more likely to perceive “multiplying” effects based on their identities. Thus, the same man who identifies as queer, Black, and having cerebral palsy may perceive an experience as discriminatory due to the combined effects of his gender identity, racial identity, and physical ability. Therefore, the experience of discrimination should be evaluated by exploring how these identities intersect and affect the individual [18].

This study assumes an intersectional model of perceptions of discrimination, as it does not differentiate the reasons participants attribute to discrimination. Certain aspects of identity can affect performance on objective assessments. Thus, if individuals have intersectional demographic characteristics and are primed with a stereotype they can internalize, such as age or economic status, they may perform worse on cognitive assessments due to internalized beliefs about task performance [42]. In one study, older adults who were told that their performance on cognitive testing would be compared with that of younger adults performed worse than older adults who were not given this information [42]. Another study found that those with low income performed worse on math and English ability tests when exposed to stereotypes stating that low-income participants perform worse on testing [43]. Objective experiences of discrimination may not always be perceived by the individual; therefore, it is important to differentiate between objective and perceived experiences, as perceived experiences are predictive of multiple life outcomes.

### 1.7. Perceived Control over Cognitive Aging and Executive Function

Oftentimes, individuals strive to live value-consistent lives, which reduces cognitive dissonance. This means that individuals with strong beliefs tend to act in ways that seem consistent with those beliefs. For example, one belief is that one has control over how they age and that one’s cognitive ability affects how they navigate and perform memory tasks. These beliefs and actions can be influenced by stereotypes and control beliefs, such as self-efficacy and locus of control.

Control beliefs affect individuals’ performance on memory tasks differently, with older adults’ control beliefs being more predictive of performance on memory tasks compared to young adults [44]. These beliefs affect older and younger adults differently, perhaps because individuals rate them differently. Younger adults may not see them as relevant to their current situation, while older adults are more likely to do so and thus have a stronger reaction. Overall, perceived control tends to be lowest in young and older adulthood while remaining stable in middle adulthood [10]. Perceptions of control tend to decline in older adulthood, which is theorized to be due to age-related limitations, such as physical health limiting the ability to perform physical activity or a lack of social resources. Therefore, older adults with more stressors may not be as affected by specific aging-related control beliefs because they are outweighed by their other pressing concerns. This highlights the complex interplay between age, control beliefs, stress, discrimination, and memory performance, suggesting a need for further research to fully understand these nuanced relationships. Experiences of discrimination, such as ageism, have been connected to decreased perceptions of control, and systemic issues that lead to discrimination undermine mastery and do not allow individuals to feel efficacious [45]. Individuals who face discrimination are also limited in their ability to control situations in which discrimination occurs [46].

This concern about decreased perception of control is especially relevant for older adults. Garstka and colleagues [47] found that older adults in the workplace were more likely to have lower levels of well-being due to ageism, compared to younger adults. Younger adults were also perceived as lower in status in their work environment compared to middle-aged adults. Their reasoning is that the permeability of group boundaries due to natural transitions, which young individuals benefit from while older individuals do not, can moderate the relation between perception of control and well-being. In other words, limited control over group membership has negative implications.

However, increased perceptions of control related to aging could also be a protective factor for older adults, as there is evidence to suggest that identifying strongly with one’s group can allow for the reassertion of control over one’s situation [47]. While discrimination that older adults face may reduce their perception of control in general, they may benefit from increased perception of aging-related control.

How much perceived control older adults have may contribute to their executive function (EF), which is already subject to decline [48,49]. Those with high levels of external locus of control were found to have lower cognitive functioning [11]. Those whose locus of control changed over time from external to internal reported significantly higher performance on cognitive functioning tasks than those with a sustained external locus of control and those whose locus of control changed from internal to external (which may occur with increased experiences of discrimination). In fact, according to Ng et al. [12], there is evidence that a sense of control mediates everyday experiences of discrimination and lower EF among older adults. This research underscores the importance of fostering a sense of agency and internal locus of control in older adults to support their cognitive health and overall well-being.

### 1.8. Discrimination, Perceived Control over Cognitive Aging, and Cognitive Function

Ly and colleagues [16] investigated how daily discrimination contributes to individuals’ perceptions of their own subjective cognition. People who reported more daily discrimination also reported lower metamemory accuracy, suggesting that those who experience frequent discrimination are at risk for cognitive decline via perceptions of their own memory [16,50]. In other words, those who experience discrimination may have more doubts about their own memory performance, which in turn may affect behaviors related to protecting cognitive function (e.g., problem-solving, trying new things) due to a lack of belief in one’s own abilities. Another study by Ng et al. [12] supported this theory. Ng et al. [12] reviewed how daily discrimination experiences affect EF via perceptions of control, including mastery (i.e., self-efficacy) and perceived constraints (i.e., the sense of uncontrollability of circumstances), in middle-aged and older adults. Ng et al. [12] analyzed data from the Midlife Development in the United States (MIDUS 2), a nationally representative study that assessed psychosocial factors and cognition among middle-aged and older adults and found that an overall sense of control mediates the relation between perceptions of discrimination and executive function, with more perceived discrimination related to less perceived control and worse executive function.

Both perceived constraint and mastery are mechanisms by which perceptions of discrimination affect executive function, with more perceived discrimination relating to less mastery and more perceived constraint, and less self-efficacy and more perceived constraint being related to worse performance on executive function tasks. Given the importance of differentiating between aspects of control, control beliefs specifically about cognitive aging may be more influential in understanding the relation between discrimination and executive function among older adults. The present study extends Ng et al. [12] by testing whether this mediation operates within a high-burden subgroup, caregiving grandparents, rather than in the general middle-aged and older adult population.

## 2. Current Study

This study examines the relations among perceived discrimination, perceived control over cognitive aging, and executive function in older adults, with a particular focus on caregiving grandparents. The literature review reveals an important gap in understanding how these factors interact within this unique population, which faces increased stressors and a heightened risk of cognitive decline. Previous research has established links between discrimination and cognitive health, the role of perceived control over cognitive aging in cognitive function, and the specific challenges faced by caregiving grandparents. However, the interplay of these factors, particularly within the context of Stress Process Theory and Family Stress Theory, remains underexplored. This study aims to address this gap by investigating the following hypotheses:

**Hypothesis 1.**  *Experiences of daily discrimination are negatively related to executive function.*

**Hypothesis 2.**  *Experiences of daily discrimination have a negative indirect effect on cognitive ability through perceived control over cognitive aging.*

**Figure 1:**
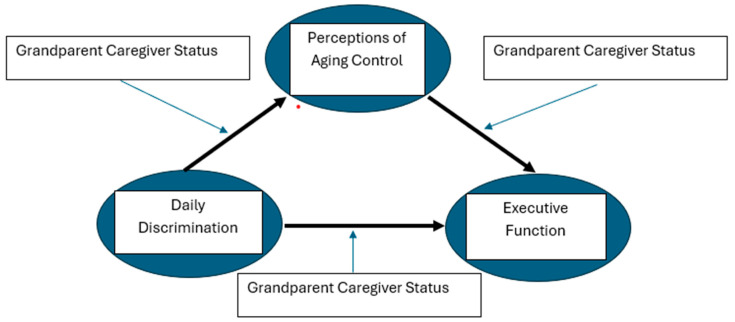
Moderated mediation model of caregiver status interactions on effects of daily discrimination on executive function via perceived control over cognitive aging.

**Hypothesis 3.**  *Grandparent caregiver status moderates the relation between experiences of daily discrimination and perceived control over cognitive aging (strengthening the relation), perceived control over cognitive aging and cognitive ability (weakening the relation), and experiences of discrimination and cognitive ability (weakening the relation); see Figure 1.*

**Hypothesis 4.**  *(Exploratory Analysis) Daily discrimination attributed to aging will not explain the relation between discrimination, perceived control over cognitive aging, and executive function due to the intersectional nature of identity.*

By examining these hypotheses, this study seeks to contribute to the literature in several ways:

1. Understanding the Impact of Discrimination: This study will provide insights into how daily discrimination, potentially exacerbated by caregiver status and socioeconomic factors, affects cognitive function in older adults. This is particularly crucial given the potential for intersectional discrimination among caregiving grandparents.
2. The Role of Perceived Control Over Cognitive Aging: Investigating the mediating role of perceived control over cognitive aging will shed light on the psychological mechanisms linking discrimination and cognitive function. This understanding can inform interventions aimed at strengthening these control beliefs and mitigating the negative effects of discrimination.
3. Focus on Caregiving Grandparents: This study addresses the unique experiences of caregiving grandparents, a population facing significant stressors that may affect their cognitive health. Examining the moderating role of caregiver status will help identify specific vulnerabilities and protective factors within this group.
4. Implications for Intervention: The findings of this study will have practical implications for developing targeted interventions and support systems to promote cognitive health and well-being in caregiving grandparents. This may include strategies to address discrimination, strengthen perceived control over cognitive aging, and provide resources to alleviate caregiver burden.

Ultimately, this study will contribute to a more nuanced understanding of the complex interplay between discrimination, perceived control over cognitive aging, and cognitive function in older adults, particularly within the understudied population of caregiving grandparents.

## 3. Materials and Methods

The current study uses data from the Midlife in the United States (MIDUS) study [51]. MIDUS is a longitudinal study of behavioral, psychological, and social factors that affect health and well-being among middle-aged adults in the United States, beginning in 1995. The MIDUS study has used a variety of recruitment strategies, including random sampling, stratified sampling, and oversampling of minority groups. The study has also included a longitudinal component, which has allowed researchers to track changes in health and well-being over time.

### 3.1. Participants

The current sample focused on participants in MIDUS 3, the third wave of the MIDUS survey. Participants were surveyed via telephone for cognitive interview questions and via a self-administered online survey for the remaining items. The analytic sample comprised 1326 participants with complete data on all study measures (166 caregiving and 1160 non-caregiving grandparents); these were drawn from the 1825 grandparents who answered the caregiving item (234 caregiving and 1591 non-caregiving). Demographic information on age, sex, race, education, and income was collected in the survey (see Table 1). Participants were majority female (56%) and White (87%; race was coded from all racial-origin fields, with about 4% identifying as multiracial, and Hispanic ethnicity was assessed separately, about 3%). The caregiving and non-caregiving groups did not differ significantly on race or ethnicity. Educational attainment was distributed across high school or less (31%), some college or an associate degree (32%), and a bachelor’s degree or higher (37%). Caregiving grandparents had lower attainment than non-caregivers. Participants were aged 43 to 93 (Mage = 67, SD = 9), with an average pretax income of approximately $52,000. Caregiver status was assessed with the items “Are you a grandparent? That is, do any of your children have a biological, adopted, step, or foster child?” and “For various reasons, grandparents sometimes take on a major responsibility for raising a grandchild. Have you ever had major responsibility for your grandchild or grandchildren for 6 months or more?” Because MIDUS records only whether a respondent had major responsibility for a grandchild for six months or more, it does not distinguish custodial, co-residing, or part-time caregiving, nor the intensity or continuity of care. We treat this heterogeneity as a limitation and recommend that future studies assess these dimensions directly.

### 3.2. Measures

#### 3.2.1. Discrimination

Experiences of discrimination were assessed with the Everyday Discrimination Scale [14], a 9-item self-report questionnaire that assesses daily experiences of discrimination with questions such as “You are treated with less courtesy than other people.” Responses are recorded on a Likert scale from 1 (often) to 4 (never). Scale items are summed and then reverse-coded, so higher scores indicate more daily experiences of discrimination. Scale reliability is high among ethnoracially marginalized individuals (α > 0.74; ref. [52]).

#### 3.2.2. Executive Function

Executive function was assessed with the Stop and Go Switch task (SGST; ref. [53]), Digits backward span, category fluency, number series, and backward counting, which were administered in the MIDUS 3 cognitive battery over the telephone. The SGST assesses inhibitory control and task switching by requiring participants to vocally state a congruent response to visual stimuli and later to switch to a task that requires stating an incongruent response to visual stimuli, which requires inhibitory control. SGST demonstrates convergent validity with the BOLOS task-switching task as expected (r = 0.52, *p* < 0.001) and with Trails B (r = 0.41, *p* < 0.001) [54]. The Brief Test of Adult Cognition by Telephone (backward digit span, category fluency, and number series) demonstrates convergent validity with associated Boston cognitive factors, with ranges from 0.42 to 54 [54]. Executive function was converted to z-scores for analysis.

#### 3.2.3. Perceived Control over Cognitive Aging

Personality in Intellectual Aging Context [55] is a 9-item self-report measure assessing older adults’ beliefs and control over their intellectual aging, with items such as “It is inevitable that my intellectual functioning will decline as I get older.” Responses range from 1 (agree strongly) to 7 (disagree strongly), and the mean of the item responses is calculated for an overall Personality in Intellectual Aging Context score, with higher scores indicating stronger beliefs about aging-related control. Scale reliability is high (α = 0.75). Negatively worded items were reverse-coded so that higher scores indicate stronger perceived control over cognitive aging.

### 3.3. Statistical Analysis Plan

IBM SPSS Statistics (version 29) and SPSS Hays Process Macro were used for data analysis. A linear regression analysis was conducted to examine the relation between daily discrimination and perceived control over cognitive aging. A mediation analysis using Model #4 was conducted to determine whether there was an indirect effect of experiences of daily discrimination on executive function via perceived control over cognitive aging. A moderated mediation analysis was conducted using Model #59 to determine the effect of caregiver status on the relations among experiences of discrimination, perceived control over cognitive aging, and executive function. Covariates of depression, education level, and income were included. Listwise deletion was used to handle missing data. We checked regression assumptions, including linearity, homoscedasticity (Breusch–Pagan and White tests), normality of residuals (Shapiro–Wilk), multicollinearity (variance inflation factors), and influential observations (Cook’s distance). Indirect effects were estimated with bias-corrected bootstrap 95% confidence intervals based on 5000 resamples, and we report the index of moderated mediation [56]. Because listwise deletion can introduce bias, we repeated the models using multiple imputation (chained equations, 40 imputations) as a sensitivity analysis. Results were consistent.

## 4. Results

### 4.1. Descriptive Statistics and Correlations

Correlation and *t*-tests were run to confirm previous research findings and determine differences between caregiving grandparents and non-caregiving grandparents for variables of interest. Correlations among variables of interest (executive function, perceived control over cognitive aging, caregiving grandparent status, and daily discrimination experiences) are presented in Table 2. As expected, daily discrimination experiences were negatively associated with executive function (*p* < 0.05), negatively associated with perceived control over cognitive aging (*r* = −0.113, *p* < 0.05), and negatively associated with caregiving grandparent status (*p* < 0.05), indicating that those who are not caregiving grandparents are less likely to report daily experiences of discrimination. Perceived control over cognitive aging was positively associated with executive function (*r* = 0.315, *p* < 0.05).

As seen in Table 3, caregiving and non-caregiving grandparents’ executive function scores did not differ significantly, nor did reports of perceived control over cognitive aging. Caregiving grandparents reported significantly more experiences of discrimination (M = 13.20) than non-caregiving grandparents (M = 11.97; t = 3.33, *p* = 0.001).

### 4.2. Mediation Analyses

**Figure 2:**
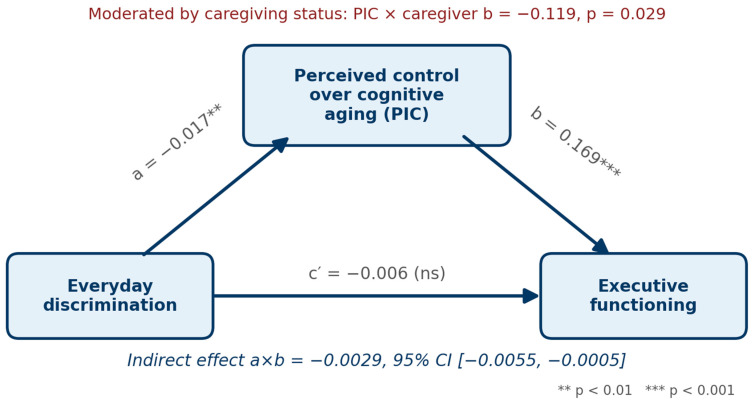
Mediation of effects of daily discrimination on executive function via perceived control over cognitive aging. *Note*: Values are beta coefficients. The direct path from daily discrimination to executive function was nonsignificant.

Using SPSS and the PROCESS macro, we tested the three hypotheses with a linear regression, a simple mediation model, and a moderated mediation model. A linear regression predicting executive function from daily discrimination was significant (b = −0.016, *p* < 0.01), supporting Hypothesis 1. A simple mediation model (PROCESS Model 4) was run with covariates of education, depressive affect, and income (see Figure 2). The indirect effect of discrimination on executive function via perceived control over cognitive aging was significant (b = −0.0029, bias-corrected bootstrap 95% CI [−0.0055, −0.0006]), and the direct effect was nonsignificant (b = −0.006, 95% CI [−0.015, 0.003], *p* = 0.17), consistent with full mediation and supporting Hypothesis 2. The constituent paths were each significant: the a-path from discrimination to perceived control (b = −0.017, 95% CI [−0.030, −0.004]) and the b-path from perceived control to executive function (b = 0.169, 95% CI [0.133, 0.205]). These effects are small in magnitude, so we interpret them as associative and do not infer clinical significance.

### 4.3. Moderated Mediation

**Figure 3:**
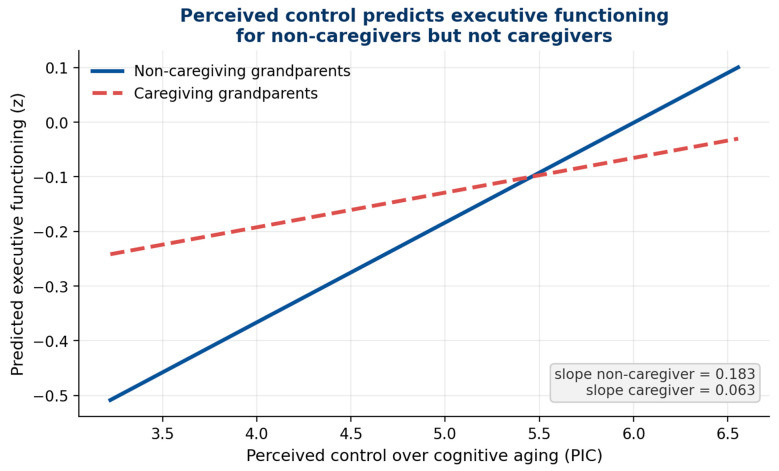
Simple slopes of perceived control over cognitive aging on executive function by caregiving status. Perceived control is positively associated with executive functioning among non-caregiving grandparents, with a markedly flatter slope among caregiving grandparents.

A moderated mediation model (PROCESS Model 59) was run with covariates of education, depressive affect, and income to test Hypothesis 3. A significant interaction emerged between perceived control over cognitive aging and caregiver status in predicting executive function (b = −0.12, *p* = 0.03), such that the positive association between perceived control and executive function was weaker among caregiving grandparents. Hypothesis 3 was thus partially supported: caregiver status did not interact with daily discrimination in predicting perceived control over cognitive aging (*p* = 0.60) or executive function (*p* = 0.46). Regression assumptions were satisfactory, with all variance inflation factors below 1.2 and no influential observations. The interaction remained significant under multiple imputation for missing data (b = −0.14, *p* = 0.008). It was attenuated to nonsignificance, however, when an expanded covariate set (age, sex, race, marital status, employment, self-rated health, chronic conditions, and social support) was added (b = −0.09, *p* = 0.085), so the moderation should be interpreted with some caution. Because the interaction was sensitive to model specification, we treat this moderating role of caregiving as preliminary and in need of replication. Figure 3 plots the simple slopes of perceived control over cognitive aging on executive function by caregiving status.

### 4.4. Exploratory Analysis

An exploratory analysis restricted to participants who attributed their experiences of discrimination to aging was run with the same model. This model produced nonsignificant results, meaning that experiences of discrimination attributed to aging were not directly or indirectly (via perceived control over cognitive aging) predictive of executive function. This supports an intersectional approach to understanding how discrimination influences cognitive functioning, rather than a unidimensional one.

## 5. Discussion

This study examined the relations among perceived discrimination, perceived control over cognitive aging, and executive function in older adults, with a particular focus on caregiving grandparents. The purpose of this study was to determine the effect of caregiver status on perceived discrimination, perceived control over cognitive aging, and executive functioning among older adults. Discrimination is a psychosocial stressor related to adverse physical and mental health outcomes, including cognitive functioning such as executive function. The results of this study provide additional support for the influence of perceived control over cognitive aging on executive function and offer valuable insights into the complex interplay of these factors and their impact on cognitive health in this understudied population. As such, this study provides further support for the application of Stress Process Theory and Family Stress Theory to caregiving grandparents. Caregiving grandparents show differences in stress-related outcomes (i.e., cognition), including the relation between perceived control over cognitive aging and executive function. This relation was not observed between discrimination and perceived control over cognitive aging, nor between discrimination and executive function. Importantly, the present findings suggest a meaningful boundary condition in this process: perceived control over cognitive aging did not function as a protective factor for caregiving grandparents. Although this resource buffered the negative effects of discrimination on executive function among non-caregivers, it failed to confer similar benefits in the context of caregiving. This pattern indicates that chronic caregiving-related stress may attenuate or disrupt otherwise protective psychosocial mechanisms.

### 5.1. Discrimination and Executive Function

Consistent with Hypothesis 1, we found that experiences of daily discrimination were negatively related to executive function in older adults. This finding aligns with previous research showing the detrimental effects of discrimination on cognitive health (e.g., [15]). Discrimination can act as a chronic stressor, leading to physiological changes that negatively affect brain structure and function, particularly in areas associated with executive control (e.g., [57]).

### 5.2. The Mediating Role of Perceived Control over Cognitive Aging

Our results supported Hypothesis 2. Perceived control over cognitive aging mediated the relation between daily discrimination and executive function. This pattern is consistent with the possibility that experiences of discrimination are associated with lower perceived control over cognitive aging, which is in turn associated with poorer cognitive performance. Perceived control over cognitive aging thus appears to be a psychological correlate linking discrimination to cognitive health. Interventions that bolster these control beliefs, such as those focused on self-efficacy and coping, may be worth examining as ways to offset the cognitive correlates of discrimination.

### 5.3. Caregiving Grandparents and Cognitive Function

Hypothesis 3, which posited a moderating role of grandparent caregiver status, was partially supported. While we did not find evidence that caregiver status moderated the relation between discrimination and perceived control over cognitive aging, or between discrimination and executive function, we observed a significant interaction between perceived control over cognitive aging and caregiver status in predicting executive function. Specifically, the relation between perceived control over cognitive aging and executive function was stronger for non-caregiving grandparents than for caregiving grandparents. This finding suggests that the potential cognitive benefits of these control beliefs may be attenuated for grandparents who are actively involved in caregiving. This may be due to the increased stress and demands associated with caregiving (e.g., [40]), which could limit the cognitive resources available for using control-related strategies. These results should be interpreted cautiously. The weaker association among caregiving grandparents may partly reflect methodological factors, such as the coarse measure of caregiving status and unmeasured caregiver burden, rather than a genuine absence of benefit from perceived control. One interpretation is that caregiving grandparents experience a cumulative stress burden that exceeds the buffering capacity of individual-level cognitive appraisals such as perceived control over cognitive aging. In line with Stress Process Theory [21], the combination of primary stressors (e.g., discrimination) and ongoing secondary stressors (e.g., financial strain, role overload) may reduce the efficacy of psychological resources that are typically protective in lower-stress contexts.

Control beliefs related to aging are also implicated in executive function, as a previous study found that personal mastery and perceived constraint mediated the relation between experiences of daily discrimination and executive function [12]. However, when considering the effects of this mediator (i.e., perceived control over cognitive aging) on caregiving grandparents, this relation is no longer significant, indicating that caregivers’ reports of their own perceived control over cognitive aging are not indicative of executive function ability.

Among caregiving grandparents, perceived control over cognitive aging may not mediate the relation between discrimination and executive function because of additional stressors. Possible mechanisms affecting these control beliefs include stereotype threat due to financial difficulties and potential experiences of discrimination related to being an older adult. Thus, reporting weaker perceived control over cognitive aging may not be related to executive function, as these beliefs may also reflect doubting one’s own abilities due to the influence of discriminatory experiences. Attributing discrimination to a single experience, such as aging, does not explain the relation among experiences of discrimination, perceived control over cognitive aging, and executive function. The intersectional nature of identity may explain why this is the case. Intersectional models that explain experiences of discrimination describe the multiplying effect of various marginalized identities on perceptions of discrimination; hence, how perceptions of why discrimination occurs may have varying influences on mechanisms explaining the relation between discrimination and executive functioning [18]. Special consideration should be given to those reporting experiences of discrimination and having executive function deficits, as the mechanisms explaining this relation may be attributed to the multiplying effects of marginalized identities rather than to a single identity. We advance these as tentative explanations to be tested in future work rather than as established mechanisms.

### 5.4. Limitations and Future Directions

This study has several limitations. One limitation is the lack of detail regarding caregiving grandparent status, as caregiving grandparents reported caring for their grandchildren for varying amounts of time. Grandparents reported how many years they were responsible for their grandchildren, but it is not specified whether they were primary caregivers during this time or whether the experience was continuous over these years. Because caregiving status is the central moderator of interest, this coarse, self-reported definition is an important constraint on the main finding and may itself contribute to the null buffering effect observed among caregivers. It could be that custodial grandparents would have different outcomes on cognitive function tasks compared to grandparents who co-reside with both their children and their grandchildren or those who provide part-time care for grandchildren in the custody of their parents. Because custodial grandparents often have worse physical health and age-related declines than noncustodial grandparents among those of lower economic status (e.g., [58]), future studies should analyze the effects of perceived control over cognitive aging on executive function independently for these groups.

Additionally, future studies could benefit from including a wider range of cognitive assessments to capture different aspects of executive function. This sample is demographically rather homogeneous, as it is a majority of highly educated, White, female respondents, and thus may not be generalizable to a wide variety of custodial grandparents. In addition, there was high variability in participants’ income status. The sample used to assess experiences of discrimination, perceived control over cognitive aging, and executive function is not representative of the racial and ethnic diversity typical among caregiving grandparents. Thus, a future study that is more ethnically diverse, as well as more gender-diverse, is recommended to assess these relations and whether they can be generalized to a larger population of individuals.

In addition, this study’s analysis was cross-sectional in nature, meaning that directional assumptions, including the predictive nature of experiences of discrimination on perceived control over cognitive aging and the predictive nature of these control beliefs on executive function, are not established. Future longitudinal studies are needed to examine the dynamic interplay of discrimination, perceived control over cognitive aging, and cognitive function over time. Because the design is cross-sectional and the effect sizes are small, we interpret these relations as associative rather than causal and allow for the possibility of bidirectional relations between control beliefs and cognition [59]. The absence of a buffering effect among caregiving grandparents may also reflect methodological limitations or residual confounding, for example, unmeasured caregiving burden, rather than a true failure of perceived control. Recent longitudinal work on grandchild caregiving and cognitive functioning offers a model for the follow-up we propose [60].

### 5.5. Implications

Despite these limitations, our findings have implications for understanding and promoting cognitive health in older adults, particularly caregiving grandparents. This study highlights the negative association between discrimination and executive function and underscores the relevance of perceived control over cognitive aging. The findings suggest that strategies to reduce discrimination and strengthen perceived control over cognitive aging may be worth evaluating for cognitive health in older adults. The unique challenges faced by caregiving grandparents should be considered when developing interventions and support systems. Future research should examine specific strategies to strengthen perceived control over cognitive aging and to address the cognitive correlates of discrimination in this vulnerable population, including efforts to address discrimination, strengthen these control beliefs, and provide resources to alleviate caregiver burden.

This research adds to the expanding literature exploring the complex social and psychological influences on cognitive aging. By examining the often-overlooked group of caregiving grandparents, we offer important insights that can guide future studies and interventions to support cognitive health and well-being among this growing demographic. The results have significant implications for public health strategies aimed at maintaining cognitive function in older adults. While interventions that strengthen perceived control over cognitive aging might benefit the broader older adult population, they may not be enough for caregiving grandparents unless combined with measures to lower chronic stress, such as improved access to social support, respite care, or financial assistance. From a public health standpoint, understanding and addressing the structural and caregiving-related stressors experienced by this group is important for protecting cognitive abilities and preventing long-term decline.

## 6. Conclusions

This study highlights the negative association between everyday discrimination and executive function among older adults and underscores the relevance of perceived control over cognitive aging as a correlate of cognitive health. Among non-caregiving grandparents, stronger perceived control was linked to a weaker negative relation between discrimination and executive function, but this protective pattern was not evident among caregiving grandparents. This preliminary difference suggests that chronic caregiving demands may reduce the influence of otherwise protective control beliefs. Because the design is cross-sectional and the effects are small, the results describe statistical rather than temporal mediation and warrant further investigation. Interventions that strengthen perceived control over cognitive aging may benefit older adults broadly, but for caregiving grandparents, they may need to be paired with measures that lower chronic stress, such as respite care, social support, and financial assistance.

## Figures and Tables

**Table 1:** Participant age, highest level of education, and income by caregiver status.

	CGP	N	Mean	Std Dev
Age	Yes	166	63.94	8.54
	No	1160	67.51	9.44
Highest Level of Education (median [IQR])	Yes	166	6 (1–2 yrs college)	[5, 8]
	No	1160	7 (3+ yrs college)	[5, 9]
Pre-income Tax	Yes	166	$45,009	46,771
	No	1160	$53,331	56,929

Note: CGP = caregiving grandparent. Age and income are reported as mean (standard deviation). Education is ordinal (1 to 12 scale) and is reported as the median [interquartile range].

**Table 2:** Correlations between executive function, perceived control over cognitive aging, and perceptions of daily discrimination experiences.

	Executive Function	Perceived Control over Cognitive Aging	CGP	Daily Discrimination Experiences
Executive Function	--	0.294 *	0.003	−0.096 *
Perceived Control Over Cognitive Aging		--	0.016	−0.101 *
CGP			--	0.101 *
Daily Discrimination Experiences				--

Note: CGP = caregiving grandparent status; * denotes significant correlations at *p* < 0.05.

**Table 3:** Mean scores of executive function, perceived control over cognitive aging, and perceptions of daily discrimination experiences by caregiving grandparent status.

	CGP	N	Mean	Std Dev	*t*-Test
Executive Function	Yes	166	−0.20	0.64	0.13
	No	1160	−0.21	0.70	
Experiences of Daily Discrimination	Yes	166	13.20	4.53	3.33 *
	No	1160	11.97	3.92	
Perceived Control Over Cognitive Aging	Yes	166	4.86	0.97	0.59
	No	1160	4.82	0.97	

Note: CGP = caregiving grandparent status; * denotes significance at *p* < 0.05.

## Data Availability

The data presented in this study are openly available in MIDUS at https://midus.colectica.org/ (accessed on 12 July 2026).
